# Mitochondrial genome study in blood of maternally inherited ALS cases

**DOI:** 10.1186/s40246-023-00516-1

**Published:** 2023-07-28

**Authors:** Sarah J. Brockmann, Eva Buck, Tiziana Casoli, João L. Meirelles, Wolfgang P. Ruf, Paolo Fabbietti, Karlheinz Holzmann, Jochen H. Weishaupt, Albert C. Ludolph, Fiorenzo Conti, Karin M. Danzer

**Affiliations:** 1 Department of Neurology, University Clinic, University of Ulm, Ulm, Germany; 2 German Center for Neurodegenerative Diseases (DZNE), Ulm, Germany; 3 Center for Neurobiology of Aging, Scientific Technological Area, IRCCS INRCA, Ancona, Italy; 4 Unit of Geriatric Pharmacoepidemiology, IRCCS INRCA, Ancona, Italy; 5 Core Facility Genomics, Medical Faculty, Ulm University, Ulm, Germany; 6 Division for Neurodegenerative Diseases, Neurology Department, University Medicine Mannheim, Heidelberg University, Mannheim, Germany; 7 Section of Neuroscience and Cell Biology, Department of Experimental and Clinical Medicine, Università Politecnica delle Marche, Ancona, Italy

**Keywords:** ALS, Mitochondrial DNA, MitoChip, Blood, Maternal inheritance

## Abstract

**Background:**

ALS is a heterogeneous disease in which different factors such as mitochondrial phenotypes act in combination with a genetic predisposition. This study addresses the question of whether homoplasmic (total mitochondrial genome of a sample is affected) and/or heteroplasmic mutations (wildtype and mutant mitochondrial DNA molecules coexist) might play a role in familial ALS. Blood was drawn from familial ALS patients with a possible maternal pattern of inheritance according to their pedigrees, which was compared to blood of ALS patients without maternal association as well as age-matched controls. In two cohorts, we analyzed the mitochondrial genome from whole blood or isolated white blood cells and platelets using a resequencing microarray (Affymetrix MitoChip v2.0) that is able to detect homoplasmic and heteroplasmic mitochondrial DNA mutations and allows the assessment of low-level heteroplasmy.

**Results:**

We identified an increase in homoplasmic ND5 mutations, a subunit of respiratory chain complex I, in whole blood of ALS patients that allowed maternal inheritance. This effect was more pronounced in patients with bulbar onset. Heteroplasmic mutations were significantly increased in different mitochondrial genes in platelets of patients with possible maternal inheritance. No increase of low-level heteroplasmy was found in maternal ALS patients.

**Conclusion:**

Our results indicate a contribution of homoplasmic ND5 mutations to maternally associated ALS with bulbar onset. Therefore, it might be conceivable that specific maternally transmitted rather than randomly acquired mitochondrial DNA mutations might contribute to the disease process. This stands in contrast with observations from Alzheimer’s and Parkinson’s diseases showing an age-dependent accumulation of unspecific mutations in mitochondrial DNA.

**Supplementary Information:**

The online version contains supplementary material available at 10.1186/s40246-023-00516-1.

## Background

Amyotrophic lateral sclerosis (ALS) is a progressive neurodegenerative disease mainly affecting motoneurons. Different genetic causes have been identified ranging from disease causing monogenic mutations, risk loci that can act in an oligogenic manner as well as epigenetic alterations [1, 2]. A great variation in disease phenotype has been reported depending on the affected genes which vary broadly in their respective function. Additionally, a large heterogeneity of clinical symptoms is known even within the same affected genes [3]. Also, mitochondrial abnormalities have repeatedly been linked to ALS [4]. However, whether sequence-related alterations of the mitochondrial genome are a common trait in ALS has not been clarified to date.

The number of mitochondria per cell depends on the energy demand of the corresponding tissue. Due to their molecular function, neurons require a high amount of energy and are therefore rich in mitochondrial mass. Motoneurons, which are the most affected cell type in ALS, have been shown to be particularly susceptible to mitochondria-associated phenotypes [5]. One of the main causes for mitochondrial DNA (mtDNA) damage is reactive oxygen species (ROS) that originate during oxidative phosphorylation [6]. Due to its spatial proximity, mtDNA is particularly susceptible to ROS-induced damage and subsequent mutations. Moreover, mitochondria have a less efficient DNA repair mechanism to eliminate acquired damages [7]. Therefore, over time mtDNA alterations accumulate in long-lived cells such as motoneurons and platelets as fragments of long living megakaryocytes. These damages are thought to be more pronounced upon disease-associated mitochondrial malfunctioning as it is the case in ALS [5, 8].

In contrast to the nuclear genome, mtDNA copy numbers vary per cell and tissue. Therefore, mtDNA alterations, including point mutations and deletions, can occur to different extents. While homoplasmic mtDNA mutations have been associated with neurological disorders, heteroplasmic mutations are known to be pathogenic if they exceed a threshold [9]. Furthermore, an accumulation of low-level heteroplasmic mutations defined as a non-reference allele frequency of < 20% can have an influence on the clinical course of a disease [10].

To address the question of whether mtDNA mutations might play a role in familial ALS (fALS), mtDNA was isolated from whole blood (WB), white blood cells (WBC) and platelets (PLT) from fALS patients and the mitochondrial genome was analyzed using a mtDNA resequencing array (Affymetrix MitoChip v2.0) that allows detection of low-level heteroplasmy in addition to the conventional homoplasmic or heteroplasmic mutations. In general, maternal transmission serves as an indicator of mitochondrial involvement in the establishment of a disease. Therefore, we distinguished between fALS with a possible maternal inheritance pattern and fALS cases that do not point to a maternal inheritance pattern. As additional controls, we compared our results to healthy individuals without any known neurodegenerative background. With this, we are aiming to get a deeper insight into a possible role of mtDNA alterations acting as a disease modifier in a subgroup of ALS patients presenting with a maternal transmission of the disease.

## Results

### Mitochondrial content is unchanged in different blood cell populations of fALS patients and controls

**Fig. 1:**
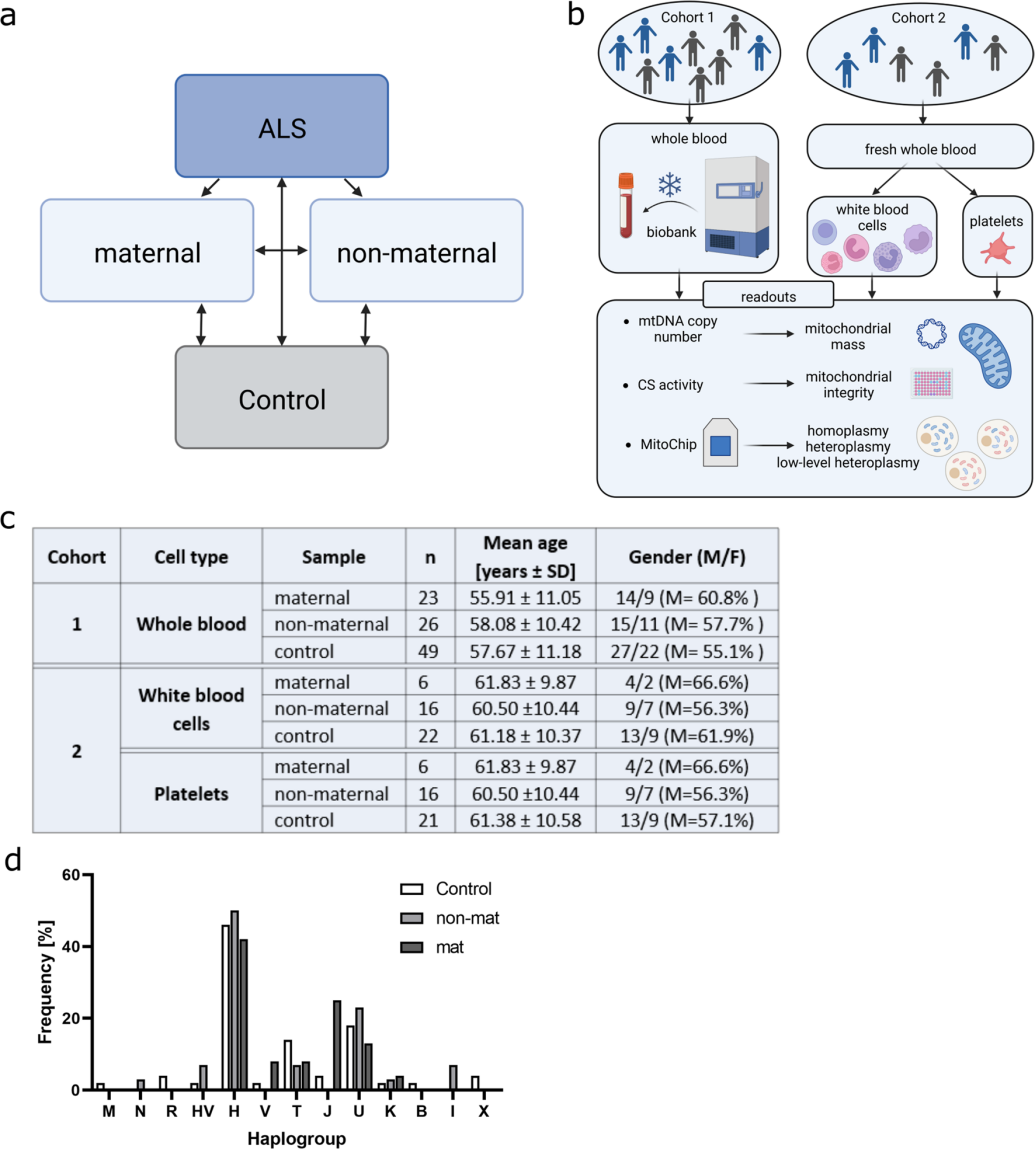
Study design and cohort characteristics. **a** ALS samples were distinguished based on pedigrees that allowed maternal or non-maternal inheritance and compared to controls. **b** Samples of cohort 1 consist of whole blood, whereas cohort 2 comprises freshly isolated platelets (PLT) and white blood cells (WBC). From each sample, mtDNA sequences and mitochondrial mass were examined using Affymetrix MitoChip v2.0 or mtDNA copy number and the citrate synthase activity determination, respectively. (Created with BioRender.com) **c** Mean age and gender of the study cohorts are listed, including the number of individuals within each group. **d** Haplogroup analysis was performed for all maternally (mat) and non-maternally (non-mat) associated ALS patients and the control group using PLT or whole blood data (Fisher’s exact test with simulated *p*-value, *p* = 0.1114)

**Fig. 2:**
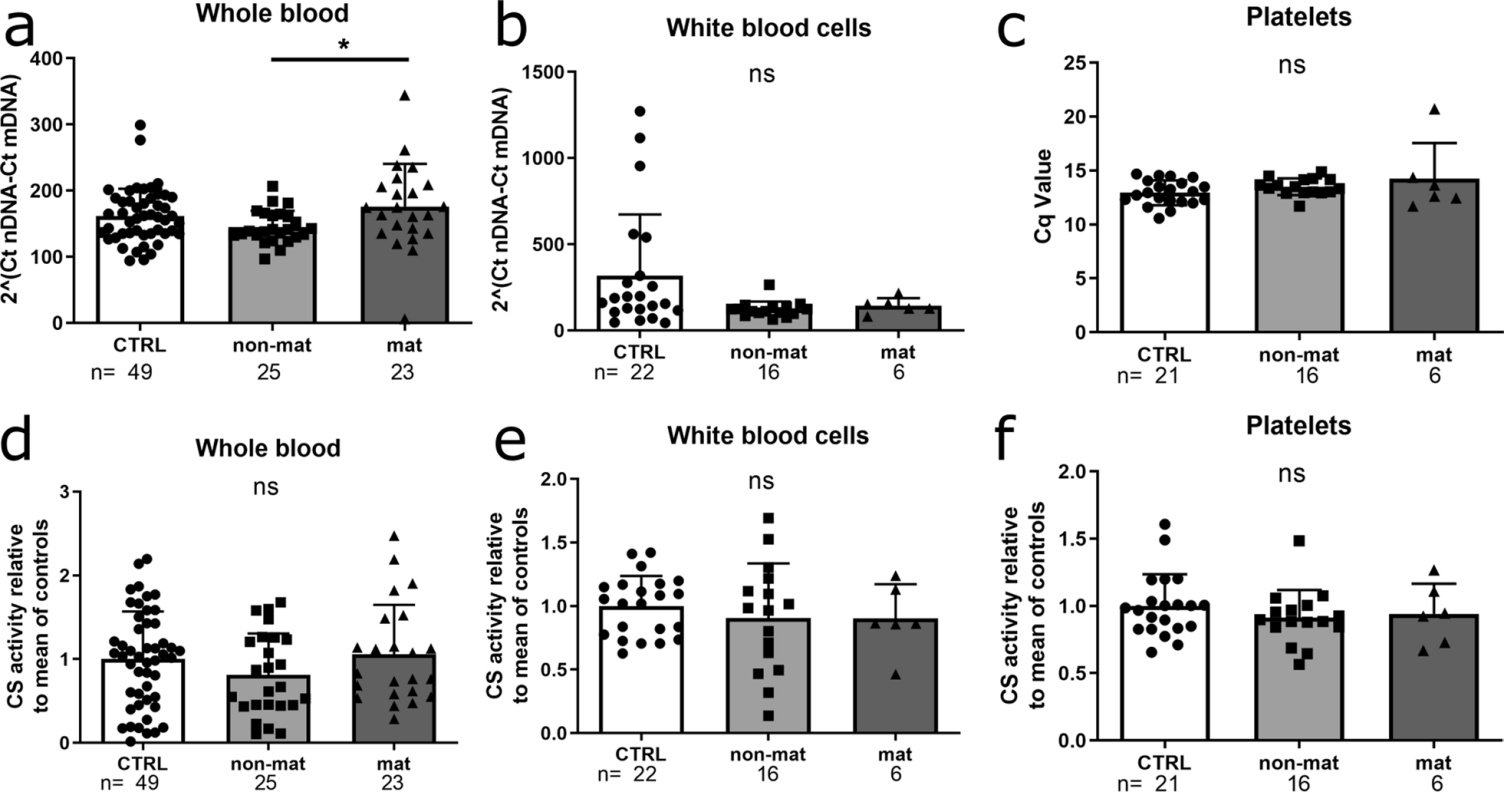
Mitochondrial mass is unchanged between ALS and controls. **a–c** Mitochondrial mass was determined by mtDNA copy number comparing the D-loop sequence to the single copy gene B2M for whole blood **a** and white blood cells (WBC), **b**, respectively. **c** mtDNA copy number of denucleated platelets (PLT) is shown as Cq value. **d–f** As a second marker for mitochondrial mass the citrate synthase activity was determined in whole blood **d**, WBC **e** and PLT **f** and displayed as (μmol*µg)/min. (Data is illustrated as mean ± SD, Kruskal–Wallis test with Dunn’s correction, *p ≤ 0.05). *CTRL* Control, *non-mat* Non-maternal ALS, *mat* Maternal ALS

To obtain a comprehensive picture of the impact of the mitochondrial genome on ALS, we set out to identify mitochondrial genome-related alterations in both, ALS cases with possible maternal inheritance as well as ALS patients without maternal association (Fig. 1a). All patients in each cohort, except for two samples, descend from different pedigrees and are therefore not genetically linked (see Additional file 1 for further information on individuals included in this study and Additional file 2 for representative pedigrees). Healthy controls were matched to patients regarding age and gender and are not genetically linked to the analyzed patients. To get an overview of mitochondrial mutations, we employed a cohort of frozen EDTA blood (whole blood, WB) of fALS patients and age- and gender-matched healthy controls that have been collected during the last decades (cohort 1). In cohort 2, white blood cells (WBC) and platelets (PLT) were isolated from freshly drawn blood samples (Fig. 1b). Mitochondrial mass was determined and total mtDNA was isolated for MitoChip experiments from all samples (Fig. 1b). Mean age and gender of the two cohorts are illustrated in Fig. 1c. To exclude sequencing differences resulting from different haplogroup distributions among the analyzed cohorts, we used homoplasmic mutations identified in platelets or whole blood to determine the respective haplogroup of each individual included in this study using mthap (https://dna.jameslick.com/mthap/). As expected, haplogroup H was predominant and overall no significant haplogroup difference was found (Fig. 1d; Fisher’s exact test with simulated *p* = value, *p* = 0.1114)**.** Although our analysis did not evidence any relationship of ALS (maternal or non-maternal) with specific haplogroups, we cannot rule out the possibility that, analyzing larger samples, an association between these pathologies and specific variants could be found. mtDNA copy number and citrate synthase (CS) activity were measured to characterize the mitochondrial content in all different sample types (Fig. 2). The mtDNA copy number is calculated based on the mitochondria encoded single copy sequence (D-loop) and relative to the nuclear encoded single copy gene (B2M). Both, mtDNA copy number (Fig. 2a–c) and CS activity (Fig. 2d–f) were unchanged in all sample types, except for a slight increase (21%) in mtDNA copy number when comparing whole blood from possible maternal versus non-maternal ALS patients (*p* = 0.0359, Kruskal–Wallis test with Dunn’s correction).

### Homoplasmic mutations in ND5 are enriched in whole blood samples of possible maternally inherited ALS patients with bulbar onset

**Fig. 3:**
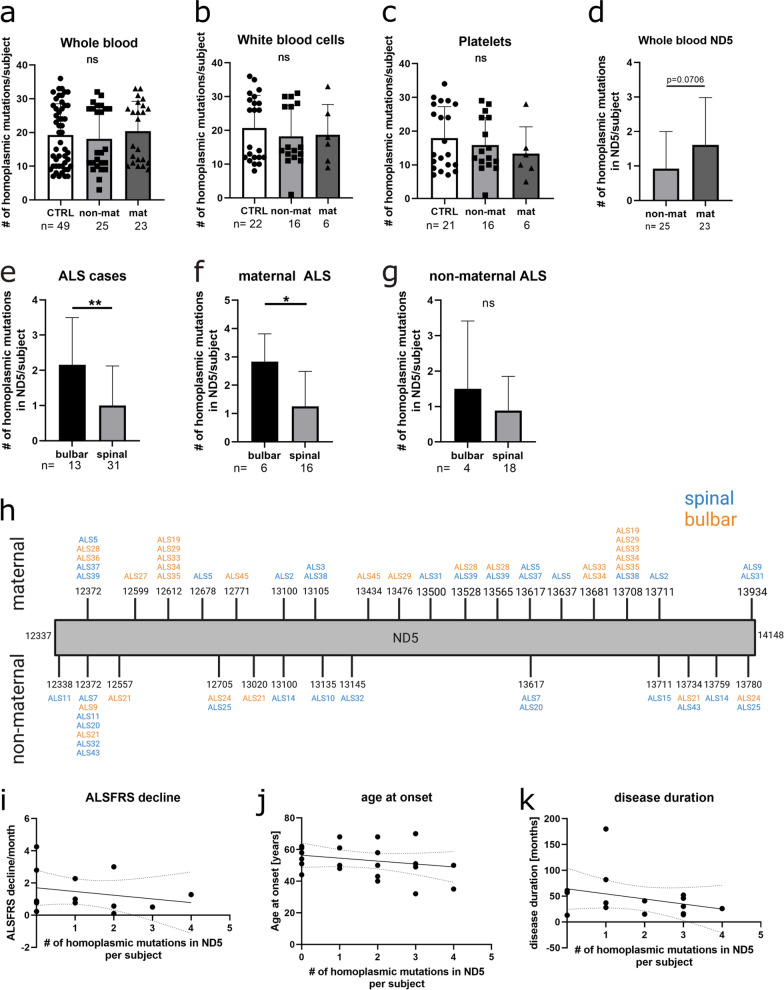
Homoplasmic mutations in ND5 are enriched in whole blood samples of maternally associated ALS patients. **a–c** For all sample types analyzed, the homoplasmic mutations per subject were counted. **d** In whole blood homoplasmic mutations in ND5 per subject showed a strong association with a possible maternal inheritance of the disease when data were analyzed per gene and compared to non-maternal samples. (Graphs show data as mean ± SD, Mann–Whitney test p = 0.0706) **e–g** The number of homoplasmic mutations in ND5 per subject is shown for all ALS cases **e**, for maternally associated ALS cases **f** as well as for ALS cases that do not allow maternal inheritance **g**, comparing ALS patients with a bulbar onset versus patients with a spinal onset (mean ± SD, Mann–Whitney test, *p ≤ 0.05, **p ≤ 0.01). **h** Graphical presentation of homoplasmic mutations on the ND5 gene in possible maternal and non-maternal patients with spinal (blue) or bulbar (orange) onset. (Created with BioRender.com) **i–k** Correlation of the amount of ND5 mutations to ALSFRS decline per month, age at onset of the disease and disease duration of the respective patients (Pearson’s or Spearman’s correlation depending on normal distribution of the data)

Homoplasmy describes a uniform composition of the mitochondrial genome in a sample. Homoplasmic mutations are thought to be inherited or occur early during development indicating that they exhibit a strong genetic component. In an unbiased approach, homoplasmic mutations were identified based on MitoChip data. After analysis, the total amount of homoplasmic mutations per subject did not differ significantly between ALS patients and controls or between ALS patients with a possible maternal versus non-maternal inheritance (Fig. 3a–c). In contrast to this general analysis, all detected homoplasmic mutations were allocated to the appropriate gene from each individual to analyze the load of homoplasmic mutations per gene. Although it did not reach statistical significance, we found a strong association in higher numbers of point mutations in ND5, a subunit of NADH dehydrogenase (complex I of the respiratory chain), in maternally linked samples compared to non-maternally linked samples when analyzing the individual point mutations by gene in whole blood (Fig. 3d; Mann–Whitney test, *p* = 0.0706). These mutations were evenly distributed among the ND5 gene as depicted in Fig. 3h. When matching the amount of homoplasmic mutations in ND5 in each patient with the according site of onset, a significant increase in bulbar versus spinal onset can be observed (Fig. 3e–g; Mann–Whitney test *p ≤ 0.05, **p ≤ 0.01). We also noticed an accumulation at positions 12,612 and 13,708 of maternally associated patients with bulbar onset (Fig. 3h). A correlation to other clinical parameters, such as ALS Functional Rating Scale (ALSFRS) decline per month, age at onset and disease duration did not show any significant effect (Fig. 3i–k, Pearson’s or Spearman’s correlation depending on normal distribution of the data).

### Heteroplasmic mutations mainly affect platelets of maternally linked ALS patients

**Fig. 4:**
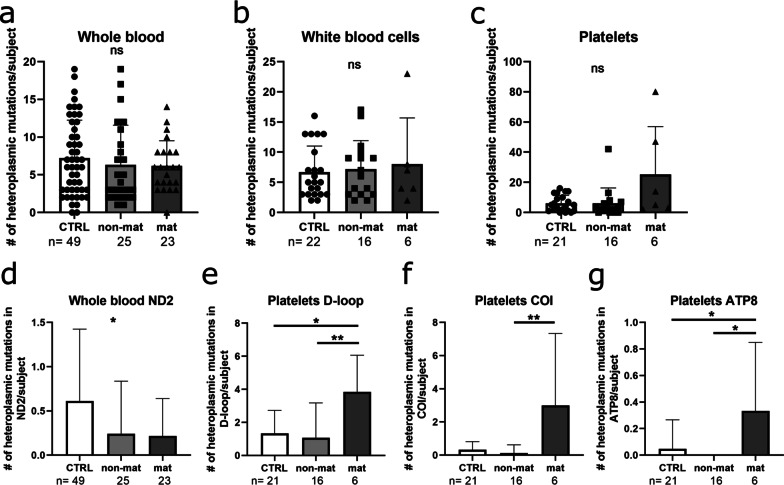
Platelets of maternally associated ALS patients show a higher heteroplasmic mtDNA mutation load. **a–c** The number of heteroplasmic mutations is shown per subject and **d**–**g** per significant gene for whole blood **a, d**, white blood cells **b** and platelets **c, e, f, g**. (Data are illustrated as mean ± SD, Kruskal–Wallis with Dunn’s correction for multiple comparison, *p ≤ 0.05, **p ≤ 0.01)

While in a homoplasmic state mutations appear in each mitochondrial genome, heteroplasmic mutations can be detected only in a fraction of the copies of mtDNA. With the GSEQ 4.1 software, heteroplasmy levels ranging from 50% down to 20% can be detected. Again, in an unbiased approach, the number of heteroplasmic mutations per subject did not show any difference in the analyzed cell populations (Fig. 4a–c). Assigning the mutations to the respective gene, heteroplasmic mutations are in general significantly increased. In ND2 of whole blood samples (Fig. 4d), as well as in the D-loop region, COI and ATP8 in platelets (Fig. 4d–g; Kruskal–Wallis with Dunn’s correction for multiple comparison, *p ≤ 0.05, **p ≤ 0.01) of possible maternal versus non-maternally linked ALS patients, the number of heteroplasmic mutations is also increased. When comparing maternally associated ALS samples with healthy controls, heteroplasmic mutations were only increased in the D-loop region and ATP8. Therefore, the maternally linked enrichment of heteroplasmic mutations in ALS patient-derived platelets indicates a susceptibility to acquire mtDNA mutations.

### Mutations are not associated to a specific nucleotide base in ALS patients

To address whether homoplasmic and heteroplasmic mutations follow a GC-AT bias, the percentage of each mutated nucleotide was calculated per subject. The comparisons among control, non-maternal and possible maternal subjects did not show any significant difference within each cell group (WB, PLT and WBC) suggesting that ALS patients and controls are not different in the bases subjected to mutation (Additional file 3; Chi–squared test).

### No increase of low-level heteroplasmy is observed in maternally associated ALS patients compared to non-maternal or healthy controls

**Fig. 5:**
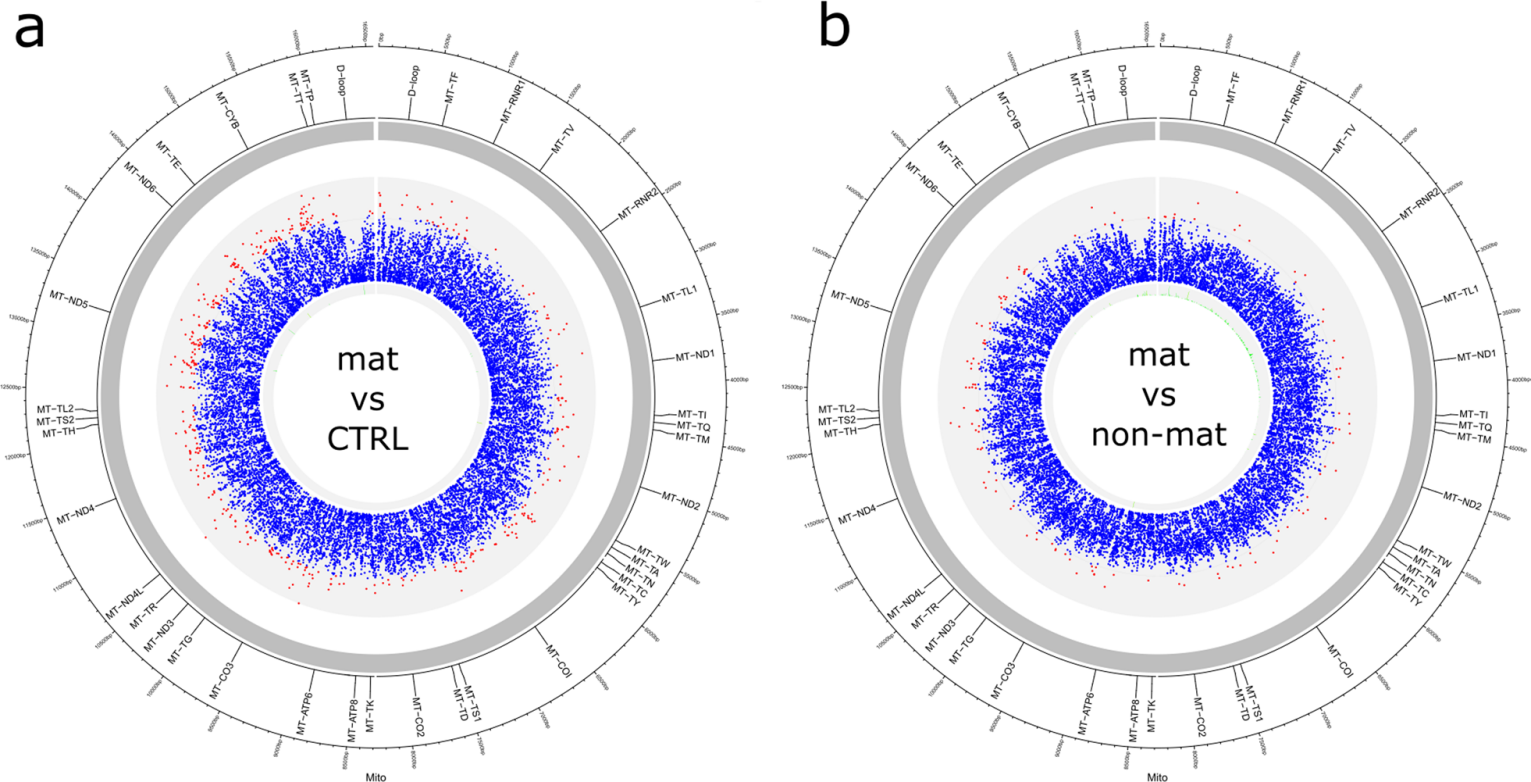
Platelets are susceptible to accumulation of low-level heteroplasmic mtDNA mutations. Graphical representation of the mitochondrial genome with gene annotations. Red dots indicate locations with significantly different (*p* ≤ 0.001, *t* test) REA values in platelets of maternally associated ALS patients compared to unaffected controls **a** or compared to ALS patients without a maternal association of the disease **b**. *bp* Base pair

Low-level heteroplasmy with a mutation load of less than 20% is an indicator of unspecific acquired mutations most likely induced by environmental factors such as ROS or by chance. To calculate low-level heteroplasmy, the contribution of the reference allele (Ratio of Expected Allele, REA) to every nucleotide position (np) is analyzed [11]. A high REA value indicates a low level of non-reference alleles, while a low value is suggestive of an increased presence of mutated alleles, in other words, of low-level heteroplasmy. The total number of significantly different np was calculated by comparing to the respective control group (Fig. 5). Especially in platelets, we saw a high number of significantly different REA values for maternally linked patients compared to control (405 np, Fig. 5a) or non-maternally linked (126 np, Fig. 5b) that are highlighted with red dots. Out of these, 65.3% and 67.3% had higher REA values in patients with a possible maternal inheritance pattern, respectively, indicating that changes in low-level heteroplasmy are associated with PLT but also that maternally associated ALS is not characterized by an increase of low-level heteroplasmy.

Overall, our data suggest that specific point mutations rather than unspecific or diffuse (low-level) heteroplasmy of the mitochondrial genome play a role in the disease course of ALS. Further, this study proposes that platelets are a vulnerable cell type regarding mtDNA stability in possible maternal ALS cases.

## Discussion

Due to the heterogeneity of clinical phenotypes of ALS, environmental risk factors have been proposed to act in combination with a genetic predisposition in a multifactorial manner. Mitochondrial dysfunction has been described as such a disease modifier. Additionally, variations in the mitochondrial genome have been associated with ALS [12]. In the current study, we analyzed different peripheral blood subpopulations of fALS patients with possible maternal and non-maternal inheritance pattern indicative of a potential mitochondrial contribution [13, 14].

We chose three different cell types to analyze the mitochondrial genome in peripheral blood. As a coarse overview, mtDNA was isolated from whole EDTA blood stored in the biobank. As an additional mtDNA source, white blood cells and platelets were separated from freshly drawn blood samples. White blood cells represent the major blood cell population and platelets were chosen because of their denucleated nature excluding a nuclear DNA background. Further, platelets derive from long-lived megakaryocytes mimicking a long life span similar to neurons.

To address mitochondrial mass in these samples, we determined mtDNA copy number and citrate synthase activity. After comparing the results to healthy controls, we did not detect a significant difference in maternally linked ALS patients in either of these two parameters. This is in line with studies in neuronal tissues. In motor neurons derived from iPSCs and human post-mortem spinal cord tissue of C9ORF72 patients, the mtDNA copy number was unaltered [15]. On the contrary, an upregulated mtDNA copy number was detected in whole blood of ALS patients carrying ALS-causing mutations in SOD1 or C9ORF72 [16] as well as a downregulated mtDNA content in post-mortem spinal cord tissue of ALS patients [17]. These contradictory results reflect the known fact that the mitochondrial content of a cell varies in different tissues and models. However, an unaltered mitochondrial mass as observed in our study might be indicative for independent effects of mtDNA mutations and mitochondrial mass in the analyzed samples.

Since the amount of mitochondrial genome varies among different cell types, mutations of mtDNA can occur to different extents. If all mtDNA copies of a sample are affected, mutations are homoplasmic. This is most likely the case if an mtDNA mutation is transmitted indicating the strong hereditary trait of homoplasmy. In our cohort, we observed an enrichment of homoplasmic mutations in ND5 in whole blood of maternally associated ALS patients compared to samples from patients with a non-maternal inheritance pattern of the disease. ND5 is a subunit of complex I of the respiratory chain and a known mutational hotspot. Complex I is a major ROS producer that releases ROS into the matrix of mitochondria in close proximity to mitochondrial genome. Therefore, a vicious cycle of an already elevated ROS level observed in ALS [18] and a further increase of ROS levels due to a malfunction of complex I induced by homoplasmic ND5 mutations might be conceivable.

For both, maternally associated and non-maternal patients, ND5 mutations seem to be distributed evenly among the whole gene. Of note, accumulations at positions 12,612 and 13,708 of maternally associated patients with bulbar onset are noticeable. According to Phylotree, both positions are also markers for haplogroup J [19]. Consequently, we found an enrichment of haplogroup J in maternally associated individuals in our study. Haplogroup J has already been associated with cognitive decline in Alzheimer’s disease patients [20] and Leber's hereditary optic neuropathy (LHON), a mitochondrially transmitted disease mainly leading to a degeneration of retinal ganglion cells [21]. Additionally, it has been shown that haplogroup J can increase the penetrance of other mtDNA mutations [22] thereby further impairing mitochondrial associated damages that play a major role in neurodegeneration. However, a possible effect of haplogroup J is population specific indicating that additional (genetic) factors are needed for a clinical manifestation of the disease [21, 23]. This is in line with our data as we also suggest a multiple hit model as underlying mechanism in maternally associated ALS patients in this study.

In this study, ALS patients with a known familial background of the disease were chosen. Therefore, the detection of mutations in mitochondrial ND5 indicate a co-existence with known ALS-causing genes as it has been shown for ND5/6 and Parkinson’s disease (PD)-related mutations in PINK1 [24]. Piccoli et al. observed a dose-dependent effect of mitochondrial mutations on onset and progression of the disease. This is in line with common knowledge that the effect of mtDNA mutations depends on a threshold meaning that up to a certain extent wildtype mtDNA can compensate for mutated alleles. Upon reaching a specific threshold, mtDNA mutations are prone to develop a detrimental phenotype [25]. In our cohort, homoplasmic ND5 mutations are enriched in whole blood samples of possible maternal ALS cases with a bulbar onset of the disease. This might indicate an additional detrimental effect of mitochondrial ND5 mutations as a bulbar onset of ALS is mostly associated with a fast progression, short survival and decreased quality of life [26]. However, a correlation to other clinical data including ALSFRS decline per month, age at onset and disease duration of ALS patients analyzed in this study did not show a relation to the amount of homoplasmic mutations. Here it needs to be noted that due to a lack of data availability, not all patients were included in the correlation. Therefore, higher sample numbers are needed to ensure that the occurrence of homoplasmic mtDNA mutations is solely associated to a bulbar onset and not dependent on other clinical parameters.

If mtDNA mutations occur to a lesser extent, wildtype and mutated copies coexist in the same cell and/or tissue (heteroplasmy). Typically, heteroplasmic mutations are either inherited or acquired during the first stages of development and accumulate in post-mitotic tissue [27]. In different blood cell types of maternally linked ALS patients, we did not observe an overall significant increase in heteroplasmic mtDNA mutations. However, in platelets, a significant increase of heteroplasmic mutations in D-loop, COI and ATP8 was detected compared to non-maternal control samples when grouping the mutations to the respective gene. Since the localization of heteroplasmic mutations seems to be randomly distributed among the whole mitochondrial genome, it seems most likely that these loci are susceptible to a higher mutation frequency [28]. In other neurodegenerative diseases such as Alzheimer’s disease, an increase of overall heteroplasmic mutations has been observed [29, 30]. ALS is associated with dysregulated metabolic activity and ROS levels [31, 32], resulting in a vicious circle of acquiring mtDNA mutations and worsening of the mutational environment. Therefore, we speculate that the contribution of heteroplasmic mutations to neurodegenerative diseases is specific to each respective disease and unlikely to be a common underlying mechanism and that the already high mutational environment in ALS can secondarily affect mtDNA by increasing heteroplasmic mutations that randomly accumulate in mutational hotspots.

By detecting eight fluorescence values for each position of rCRS (including the sense and antisense strand), the MitoChip used in this study is able to detect even low-level heteroplasmy affecting less than 20% of the analyzed mitochondrial genome. To assess mutations at these low levels, the ratio of expected allele frequency (REA) is calculated by building the logarithmic ratio of signal intensity of reference nucleotide at any position to average signal intensity of the 3 other nucleotides. Therefore, a high REA value stands for a high proportion of the reference allele at a certain position. In our maternally associated fALS cohort, we detected a significant difference of low-level heteroplasmy in some np from platelets compared to each of the control groups. Interestingly, in two-thirds of the detected nucleotide positions, REA values are higher for possible maternal ALS patients (65.3% and 67.3% in comparison to healthy controls and non-maternal fALS patients, respectively) indicating a non-relevant increase of low-level heteroplasmy in healthy controls and non-maternal fALS patients. mtDNA mutations occurring on such a low level are mainly acquired during lifespan as a result of damage accumulation. Again, in Alzheimer’s and Parkinson’s disease an increase of non-reference alleles has been observed indicating a contribution of diffuse and unspecific mtDNA alterations to the disease phenotype [33, 34]. This is contrary to our findings in ALS indicating that low-level heteroplasmy of mtDNA is not associated with maternally linked ALS.

MitoChips have been extensively used to study mtDNA mutations associated with cancer, Alzheimer’s disease, polymorphisms and rare mutations [11, 35–37]. MitoChip sequencing has been well characterized in terms of sensitivity, specificity, and accuracy in detecting homoplasmic and heteroplasmic variants, and has been demonstrated to provide reproducible results, thus making a verification procedure unnecessary [38, 39]. Other methods are available to sequence the mitochondrial genome. Comparing MitoChip results to capillary electrophoresis sequencing showed a concordance between both methods of 99.999% [40]. In particular, next-generation sequencing (NGS) technology has proven to be a reliable technique providing a quantitative estimation of heteroplasmy level, although appropriate criteria for avoiding false positives are required. Both, MitoChip sequencing and NGS show comparable performances with regards to base call accuracy [41]. Analytical tools for arrays have been honed over several years and a general consensus has emerged on the most effective methods to process data, while as of now NGS data analysis poses a big challenge in terms of the huge quantity of data and the level of sophistication necessary to analyze them.

Taken together, we found an enrichment of homoplasmic mtDNA mutations in ND5, a subunit of complex I of the respiratory chain in whole blood. Platelets seem to be a relevant blood cell population as they accumulate heteroplasmic mutations. It has been shown for other age-related neurodegenerative diseases that a randomly distributed increase of mtDNA alterations can lead to a general instability of mtDNA which is most likely caused by an “aging” effect whose impact depends mainly on constitutive baseline mitochondrial function and exposure to environmental factors [33, 34, 42]. However, in this study, we see a rather directed mutational burden affecting mostly complex I of the respiratory chain, particularly the subunit ND5 in possible maternal ALS cases with bulbar onset. Interestingly, an additional load of heteroplasmic mutations is present in platelets that derive from long-lived megakaryocytes.

To our knowledge, this is the first study analyzing mitochondrial genome changes in fALS patients presenting with a pedigree that also allows maternal inheritance. By showing that homoplasmic mutations are a common trait in maternally linked ALS, our results suggest that specific rather than diffusely distributed mtDNA mutations accumulate in ALS and might act as an additional disease modifier on top of a monogenic cause of the disease.

## Conclusions

The data generated in this study indicate a contribution of mtDNA mutations to the course of disease of fALS patients that are associated with a maternal inheritance pattern. Especially homoplasmic mutations in ND5, a subunit of complex I of the respiratory chain, are enriched in whole blood of possible maternal fALS patients with bulbar onset. Therefore, specific mtDNA mutations rather than diffusely distributed mutations that have been observed in Alzheimer’s and Parkinson’s disease suggest a direct genetic link of mtDNA mutations detected in different blood cell populations to maternally associated fALS.

## Methods

### Ethics statement

All experiments comprising human samples were performed in accordance with the declaration of Helsinki and approved by the Ethics Committee of Ulm University. All study participants gave informed written consent to participate in the study. ALS patients and healthy controls were recruited at the Universitäts- und Rehabilitationskliniken Ulm (RKU Ulm). Healthy controls were chosen to match the patient cohort’s characteristics. ALS patients were clarified as familial ALS patients due to a clear family history of the disease. Blood was drawn from familial ALS patients whose pedigrees allowed maternal inheritance and compared to ALS patients without maternal association (non-maternal) and age-matched controls (see Additional file 2 for representative pedigrees). Whole blood samples from the biobank included 27 patients carrying mutations in different ALS-causing genes (C9ORF72, SOD1, FUS or KIF5A) and 21 patients with a familial ALS history but without suspicious variants in known ALS genes. PLT and WBC were freshly isolated from 6 patients with maternal association (2 with known ALS mutations (1 × C9ORF72, 1 × SOD1), and 4 without any known mutation) and 16 patients without maternal association (6 with known ALS mutations (4 × C9ORF72, 2 × SOD1), and 10 without any known mutation). Further patient information is listed in Additional file 1.

Taken together, whole blood samples from 48 ALS patients (cohort 1) and platelet/white blood cell samples from 22 ALS patients (cohort 2) were included in this study. It needs to be noted that 15 patients from cohort 1 were also included in cohort 2, but blood was drawn at different time points. Control samples did not overlap in both cohorts. Therefore, a total of 55 ALS patients and 71 controls were analyzed in this study.

### Isolation of PLT and WBC

PLT and WBC were separated from 15 to 40 ml of fresh EDTA blood by several rounds of differential centrifugation. Finally, magnetic bead depletion of CD45^+^ cells for PLT and depletion of CD61^+^ cells for WBC using MACS® Cell Separation (Miltenyi Biotec, Hilden, Germany) cleaned up the populations, which were stored at − 80 °C for further experiments.

### DNA isolation (nuclear and mtDNA)

Total DNA of PLT, WBC or WB samples was isolated using the QIAamp DNA Mini and Blood kit (Qiagen, Hilden, Germany).

### mtDNA copy number

Genomic and mtDNA were isolated from frozen PLT or WBC pellets or from 500 µl EDTA blood using the QIAamp DNA Blood Mini Kit (Qiagen, Hilden, Germany). Levels of the nuclear encoded single copy gene beta-2 microglobulin (B2M) and of the mitochondrial displacement loop (D-loop) were determined in triplicates by qPCR. The mtDNA copy number for whole blood and white blood cells was calculated using the formula (2^(Ct nDNA-Ct mDNA)), whereas for PLT the Cq value is reported due to the lack of nuclear DNA [43].

### Citrate synthase activity assay

Mitochondrial citrate synthase (CS) activity was determined in triplicates by detecting the absorbance of thionitrobenzoic acid (TNB) at a wave length of 412 nm. A porcine heart CS standard was used as reference in each experimental run. The CS activities are reported in (μmol*µg)/min [43].

### MitoChip v2.0 Affymetrix

Analysis of mtDNA sequence variations was done using GeneChip™ Human Mitochondrial Resequencing Arrays 2.0 (MitoChip v2.0) [44]. For processing of the arrays, the GeneChip® CustomSeq® Resquencing Array Protocol version 2.1 was used with the following modification: instead of using long-range PCR, mtDNA was amplified using the REPLI-g mitochondrial DNA kit (Qiagen Hilden, Germany), as it has been described for MitoChip experiments before [45], and purified using Agencourt® AMPure® XP magnetic beads (Qiagen Supplementary Protocol, REPLI-g mitochondrial DNA kit). After purification, 270 ng of mtDNA was fragmented and labeled using the GeneChip® Resequencing Assay Kit (Affymetrix, now part of ThermoFisher Scientific). After hybridization, MitoChip v2.0 arrays were washed and stained in a Fluidics Station 450 before being scanned in Affymetrix GeneChip Scanner 3000 7G. CEL files were acquired by GeneChip® Operating Software 1.4 (GCOS 1.4.0.036) and analyzed with GeneChip® Sequence Analysis Software (GSEQ 4.1). Complete microarray data are available at Gene Expression Omnibus (GEO accession number: GSE211250).

### MitoChip v2.0 data analysis

GSEQ 4.1 uses an objective statistical framework for the assignment of a base call to each nucleotide position (np) complying with the quality score as defined in the resequencing algorithm. We set ‘‘model type’’ at diploid to enable the detection of heteroplasmy and ‘‘quality score threshold’’ at 3 to provide the best base calling accuracy and rate. The output files utilized for this study were the Single Nucleotide Polymorphism (SNP) View and Probe Intensity Files that provide, the base call and the values of fluorescence intensity of the four bases for each np for both sense and antisense strands, respectively. SNP are classified as homoplasmic, heteroplasmic, or no-calls. The homoplasmic and heteroplasmic mutations are defined in comparison with the revised Cambridge Reference Sequence (rCRS) while a no-call is given when the quality score for the np investigated is below the threshold defined in the algorithm. The values of Probe Intensity Files were used to calculate REA (Ratio of Expected Allele) index, which is defined as the log ratio of the signal intensity of the reference nucleotide, as indicated in the rCRS, to the average signal intensity of the other three alleles from the sense and antisense strand [11]. A high REA value indicates a prevalence of the reference nucleotide, whereas a low value suggests a significant contribution of the other three nucleotides. The number of homoplasmic and heteroplasmic mutations was counted in each subject or gene and averaged for each group. Analyses were performed by Kruskal–Wallis test with Dunn’s correction for multiple comparisons. REA values were calculated for the 16,544 np in every subject, and the np means were determined in each of the three patient groups. Before comparing mean values of each np, we applied the Shapiro–Wilk test to examine the distribution of the data and, depending on the result, we used Student’s *t* test (normal distribution) or Mann–Whitney test (not-normal distribution) comparing the groups two-by-two. Significance level was set at *p* ≤ 0.001.

### Base prevalence

Homoplasmic and heteroplasmic mutations were counted for each base (a, c, g or t), and the mean number per subject was calculated. Then, as the number of the four bases is different in the mtDNA sequence, we determined the percentage of mutations on the total number of each base. The comparisons among the groups (CTRL, maternal and non-maternal ALS) were performed with Chi-squared test.

### Haplogroup analysis

Haplogroup analysis was performed on the basis of PLT or whole blood data from each subject using mthap (https://dna.jameslick.com/mthap/) [19]. For statistical analysis, Fisher’s exact test with simulated p-value was applied. Further information on haplogroup distribution in the analyzed cohort can be found in Additional file 1.

### Statistical analysis

If not stated otherwise, statistical analysis was performed using Graph Pad Prism 9. All data were tested for normal distribution by Kolmogorov Smirnov test. For comparison of two samples, Mann–Whitney test was chosen. Several groups were analyzed by Kruskal–Wallis test with Dunn’s correction for multiple comparison testing. Bar graphs show the mean values ± SD. For correlation of clinical data, Pearson correlation coefficients or nonparametric Spearman correlation depending on normal distribution of the data were applied.

## Supplementary Information

**Additional file 1 **This excel sheet contains detailed information on all subjects (patients and controls) analyzed in this study including age and sex and disease parameter for ALS patients.

**Additional file 2 **In this figure, representative pedigrees of families with a maternally associated **a**, **b** or non-maternal **c**, **d** inheritance are shown. The respective individual included in this study is marked with a red arrow.

**Additional file 3 **Homoplasmic and heteroplasmic mutations do not show changes in base prevalence. Mean numbers of homoplasmic and heteroplasmic mutations per subject were calculated as percentage of mutations out of the total number for each base in the mtDNA molecule. To analyze a possible mutational bias concerning a specific base, a Chi-squared test was performed in different cell types.

## Data Availability

The dataset generated during the current study is available in the Gene Expression Omnibus repository, (GEO accession number: GSE211250) https://www.ncbi.nlm.nih.gov/geo/query/acc.cgi?acc=GSE211250.

## Abbreviations

ALS: Amyotrophic lateral sclerosis
B2M: Beta-2 microglobulin
D-loop: Displacement loop
fALS: Familial amyotrophic lateral sclerosis
mtDNA: Mitochondrial DNA
PLT: Platelets
rCRS: Revised Cambridge Reference Sequence
WB: Whole blood
WBC: White blood cells
