# Supplementary figures and images for: Mitochondrial genome study in blood of maternally inherited ALS cases
Source: Hum Genomics. 2023 Jul 28;17:70. doi: 10.1186/s40246-023-00516-1 (PMC10375681; doi:10.1186/s40246-023-00516-1)

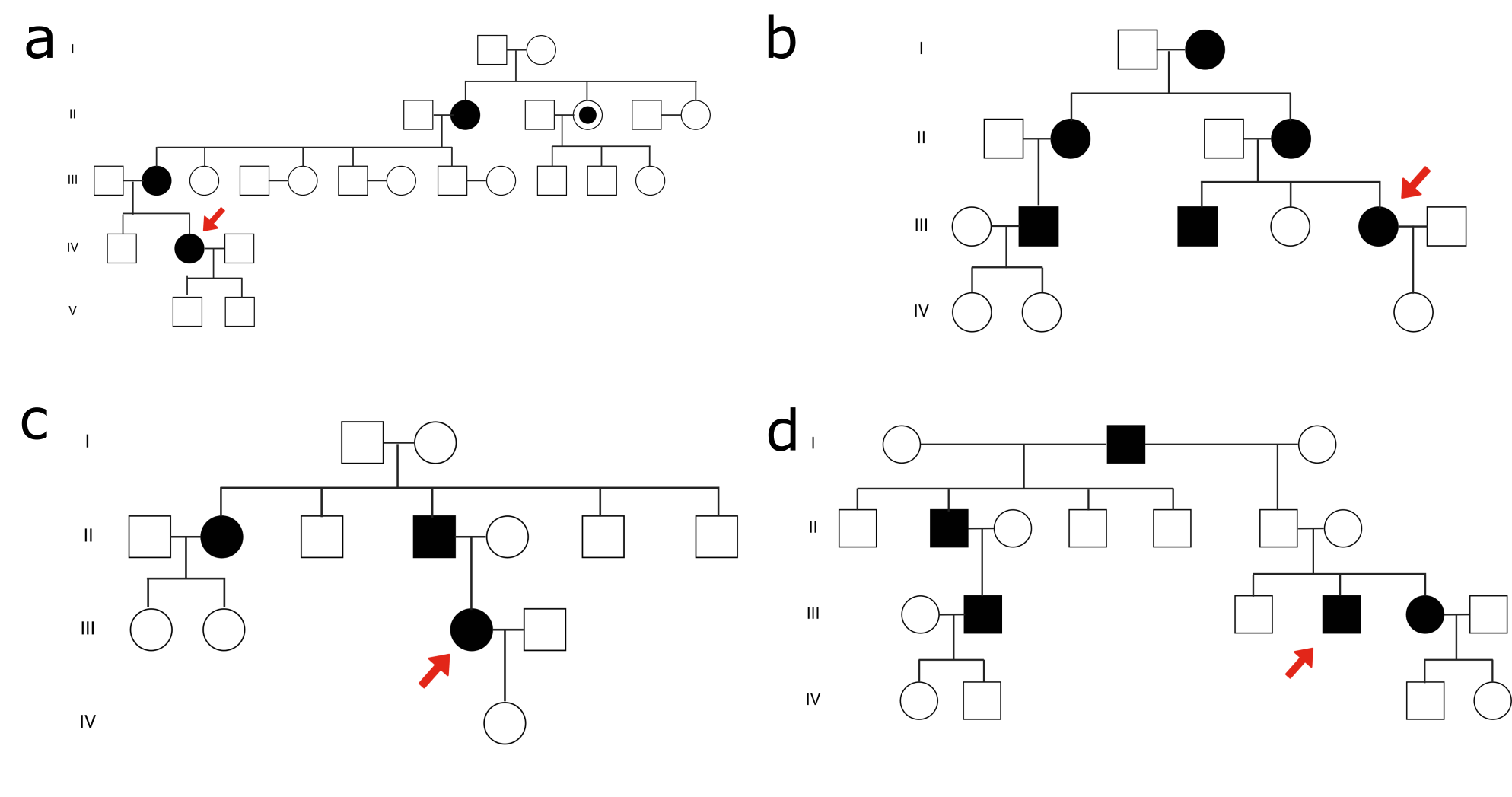

Supplement: Supplementary file 2 — Additional file 2 In this figure, representative pedigrees of families with a maternally associated a, b or non-maternal c, d inheritance are shown. The respective individual included in this study is marked with a red arrow. [file 40246_2023_516_MOESM2_ESM.tiff]

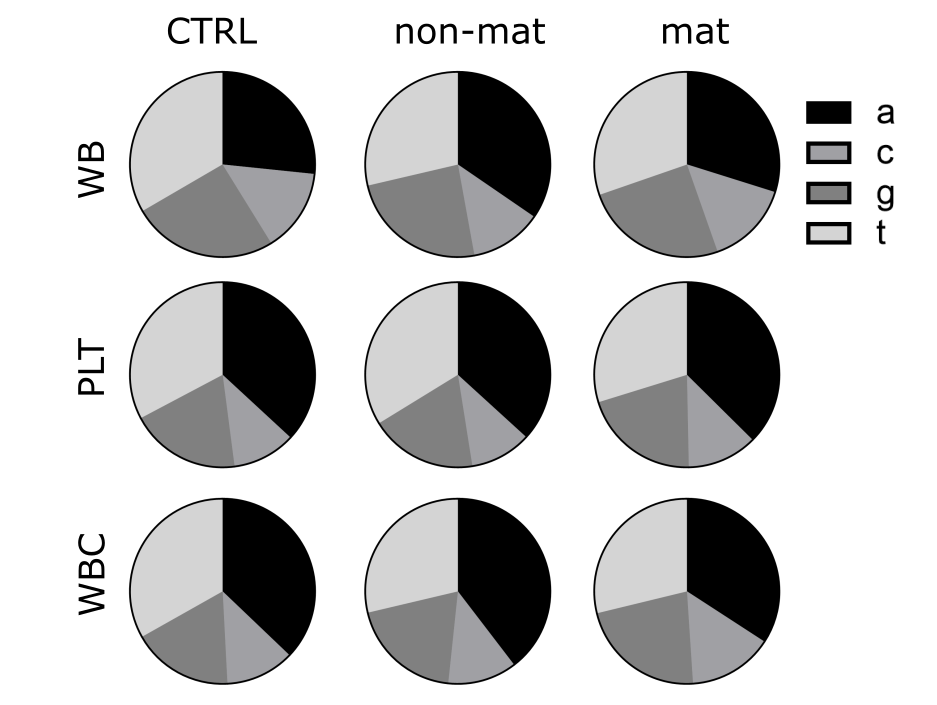

Supplement: Supplementary file 3 — Additional file 3 Homoplasmic and heteroplasmic mutations do not show changes in base prevalence. Mean numbers of homoplasmic and heteroplasmic mutations per subject were calculated as percentage of mutations out of the total number for each base in the mtDNA molecule. To analyze a possible mutational bias concerning a specific base, a Chi-squared test was performed in different cell types. [file 40246_2023_516_MOESM3_ESM.tiff]
